# The experience of effort in ADHD: a scoping review

**DOI:** 10.3389/fpsyg.2024.1349440

**Published:** 2024-06-03

**Authors:** Danika Wagner, Samantha G. Mason, John D. Eastwood

**Affiliations:** ^1^The Boredom Lab, Department of Psychology, York University, Toronto, ON, Canada; ^2^Department of Psychiatry, Sunnybrook Research Institute, Toronto, ON, Canada

**Keywords:** effort, ADHD, conscious experience, mental effort, effort preferences, self-report

## Abstract

**Background:**

Mental effort plays a critical role in regulating cognition. However, the experience of mental effort may differ for individuals with Attention-Deficit/Hyperactivity Disorder (ADHD), a disorder for which sustained mental effort ‘avoidance’ or ‘dislike’ is a criterion in the DSM. We conducted a scoping review to characterize the literature on the experiences of effort in ADHD.

**Methods:**

This systematic scoping review adhered to the Preferred Reporting Items for Systematic Reviews and Meta-analyses (PRISMA) Extension for Scoping Reviews and Joanna Briggs Methodology. PsycINFO (OVID), PsycINFO (ProQuest) and PubMed were searched for studies published in English before February 14, 2023. Studies must have included an ADHD population or a measure of ADHD symptomatology, in addition to a self-report measure of the experience of effort or the use of an effort preference paradigm. Two researchers reviewed all abstracts, and one researcher reviewed full-text articles.

**Results:**

Only 12 studies met the inclusion criteria. Several gaps and inconsistencies in the research were identified in terms of method, definitions of effort, measurements of ADHD, and sample characteristics. Moreover, the pattern of results on the experience of effort was mixed.

**Conclusion:**

Despite its diagnostic and conceptual significance, the experience of mental effort in ADHD is not well studied. Critical gaps were identified in the existing literature. A three-facet conceptualization of effort is proposed–specifically, task-elicited effort, volitionally exerted effort, and the affect associated with engaging in effort – to guide future explorations of the experience of effort in ADHD.

## 1 Introduction

Attention-Deficit/Hyperactivity Disorder is a common neurodevelopmental disorder characterized by pervasive, persistent, and impairing symptoms of inattention, impulsivity, and/or hyperactivity (Polanczyk et al., 2014; APA, 2022). Attention-Deficit/Hyperactivity Disorder (ADHD) has been the subject of considerable research, yet some diagnostic criteria have received limited research attention (Lahey et al., 1994; Matte et al., 2015). Indeed, the wording of criterion (f) for the Inattention subtype changed from “often seems unmotivated to do schoolwork or homework” to “often avoids, dislikes, or is reluctant to engage in tasks that require sustained mental effort” after the completion of the field trials for the DSM-IV (Lahey et al., 1994; APA, 2022). ‘Dislikes’ or ‘preferences’, as indicated through behavioral avoidance or direct verbal reports, involve valenced feelings and are considered affective phenomena (e.g., Scherer, 2005). Although cognitive factors may contribute to this symptom, the criterion describes an affective phenomenon. To inform clinical practice and research, the present study provides a scoping review of the existing literature on the experience of mental effort in individuals with ADHD.

Existing research on the reworded criterion (f) has examined symptom utility, which provides information on the diagnostic value of a specific symptom. Measures of symptom utility include conditional probability statistics, such as corrected positive predictive power (cPPP), which is the conditional probability of the disorder being present given the presence of a symptom corrected for limits on the maximum possible values because of extremely high or low base rates (Frick et al., 1994). Frick et al. (1994) found that criterion (f) had good cPPP (0.74) and reasonable corrected negative predictive power (0.59). Frick et al. (1994) also found that this symptom had the highest positive predictive power (uncorrected) of all inattention symptoms in the younger sample (ages 4–13) but the lowest positive predictive power in the older sample (ages 14–17). Owens and Hoza (2003) found this symptom to have good sensitivity and corrected PPP for predicting ADHD subtypes based on DSM-IV-TR criteria. Given these findings, the criterion seems to provide utility in ADHD diagnoses. However, little is known about the experience of mental effort that may drive the avoidance of tasks observed in ADHD.

The concept of mental effort has been understood and operationalized in various ways (Székely and Michael, 2021). At an abstract level, mental effort is typically conceptualized as the energy required to accomplish mental activity (Kahneman, 1973; Inzlicht et al., 2018) that is fueled by physiological arousal and activation and that acts through attentional processes (Mulder, 1986; Shenhav et al., 2017). Some mental effort models emphasize the notion that tasks elicit mental effort from an actor (Kahneman, 1973). In contrast, others focus on the volitional exertion of effort by the actor (Hockey, 20111), while others focus on the interplay between task-elicited and volitionally exerted processes (Mulder, 1986; Fairclough and Mulder, 2012; Inzlicht et al., 2018). Some models are articulated primarily in terms of the underlying cognitive processes (Hockey, 2011; Shenhav et al., 2017), while others make the experience of engaging in mental effort central to the model (e.g., Botvinick, 2007; Kurzban et al., 2013; Kurzban, 2016). The experience of engaging in mental effort has typically been conceptualized as a reflection of the sufficiency of non-controlled processes (e.g., Hockey, 2011) or the cost associated with exerting effort (e.g., Kurzban et al., 2013). However, various underlying costs have been proposed (Kurzban, 2016). Because the conscious experience of mental effort is thought to index a cost and make this cost salient to the actor, it is generally aversive and serves to motivate subsequent behavior (e.g., Kool et al., 2010; Kurzban et al., 2013; Hsu et al., 2017). That is, the experience of mental effort motivates withdrawal and avoidance and/or the actor must engage in self-control to persist with the aversive mental activity.

Theory-based distinctions between task-elicited and volitionally exerted cognitive processes and the experience of mental effort have been born out in empirical findings. For example, Mulert et al. (2007) found that self-reported task-elicited effort was proportional to objective task difficulty, while volitionally exerted effort remained consistent across difficulty levels. Additionally, Otto et al. (2014) found that ratings of task-elicited effort, compared to task difficulty ratings, were associated with increased left anterior insular cortex activation, an area of the brain that plays a significant role in emotional self-awareness. Work exploring flow, or autotelicity, highlights an extreme example of high task-elicited and low volitionally-exerted mental effort [for review, see Bruya (2010)].

**Figure 1:**
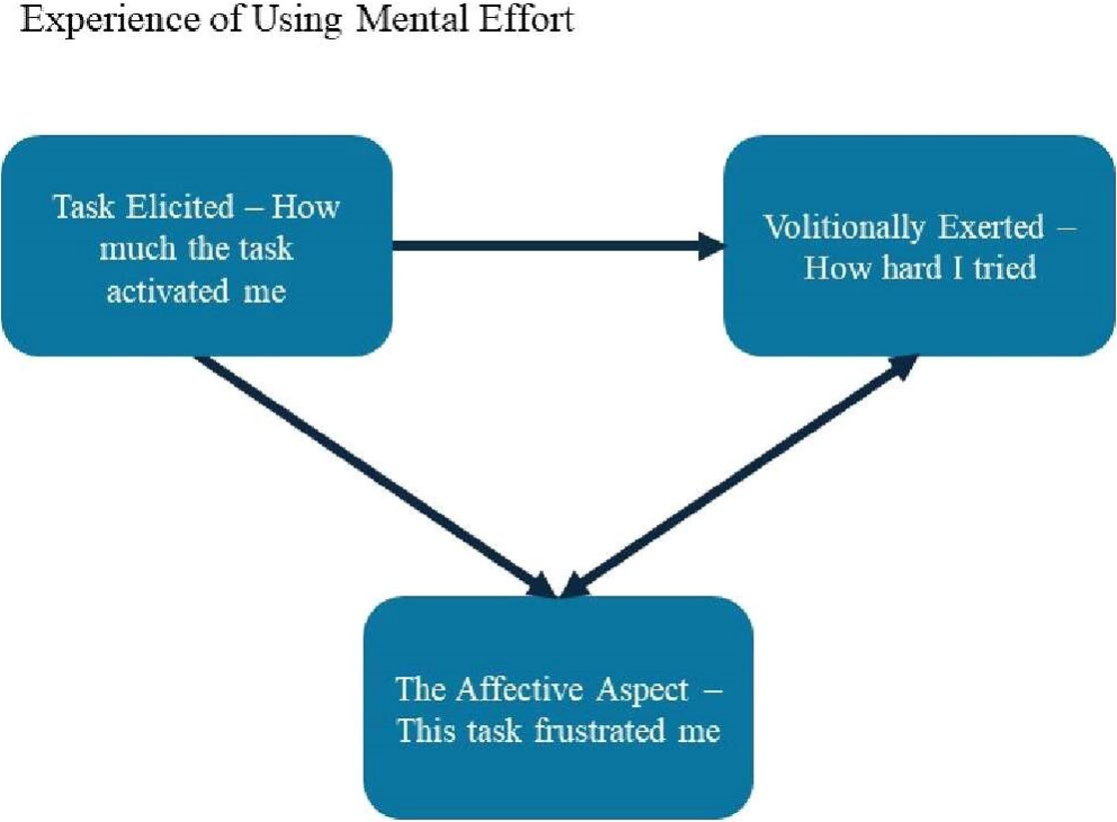
PRISMA-ScR flowchart. *Wrong study design means that the studies did not assess the conscious experience of effort.

In summary, in terms of underlying cognitive processes, it is essential to distinguish between ‘task-elicited’ mental effort and ‘volitionally exerted’ mental effort. Moreover, existing research suggests that these different components of mental effort can be experienced and distinctly reported. Finally, there is an affective feeling associated with engaging in mental effort (e.g., Kool et al., 2010; Hsu et al., 2017; Bambrah et al., 2019), and this feeling is critical to understanding avoidance of, or persistence with, cognitive tasks. See Figure 1 for a summary of these distinct aspects of mental effort.

Sergeant’s cognitive-energetic model of ADHD proposes that deficiencies in effort regulation underpin ADHD deficits (Sergeant and Oosterlaan, 1999; Sergeant, 2000, 2005). Like other theorists, Sergeant defined mental effort as the energy necessary to meet task demands. This model considers effort an energetic pool that operates as a regulatory mechanism via arousal and activation. In Sergeant’s model, effort works by inhibiting and exciting arousal and activation to promote lower-level information processing, such as encoding, search, decision preparation, and motor organization. Additionally, effort serves to regulate upper-level management of executive functioning systems. Thus, in Sergeant’s model, effort operates as an intermediary resource manager that responds to inputs from top-down and bottom-up levels of information processing. When tasks are sufficiently engaging, the task elicits effort from the individual. Problems arise when tasks are under or over-engaging, requiring volitional effort to meet task demands. Thus, the conceptual distinction between task-elicited and volitionally exerted components of mental effort is implied in Sergeant’s model of ADHD.

The experience of mental effort can be measured with multi-faceted self-report scales. These scales typically use a Likert-type rating indicating the participant’s mental or affective states on multiple dimensions. Some multi-faceted measures, like the NASA Task Load Index (NLTX), which measures the construct of workload by assessing mental, temporal, and physical demand, effort, performance, and frustration, have been used with ADHD populations (Hart and Staveland, 1988; Mies et al., 2019). While other multi-faceted scales, like the Dundee State Stress Questionnaire (Matthews et al., 1999) or the Subjective Workload Assessment Technique (Potter and Bressler, 1989), have not, to our knowledge, been used with ADHD populations.

In some research, participants have been asked to rate their level of effort or discomfort on a single scale (e.g., Zijlstra and Van Doorn, 1985; Paas and Van Merrienboer, 1993; Hsu et al., 2017). These single-item scales usually ask participants to report their volitionally exerted effort (e.g., Zijlstra and Van Doorn, 1985; Paas, 1992; Paas and Van Merrienboer, 1993; but see Hsu et al., 2017 for an exception). For example, the Paas (1992) scale asks participants to rate how much mental effort they invested in a task from one (very, very low mental effort) to nine (very, very high mental effort). Single-item scales have been used to understand the experience of effort in ADHD (e.g., Hsu et al., 2017; Brown et al., 2020). Such scales have the advantage of brevity but often fail to capture the entire conscious experience of mental effort. Moreover, single-item scales are limited psychometrically in terms of reliability and validity.

Beyond self-report methods, researchers have also used preference paradigms to infer the experience of effort in individuals with ADHD. There are several preference paradigms for studying physical and mental effort, which differ in the task demands, the type of effort they purport to measure, and the presence of reward. Tasks that measure physical effort often require repeated button presses, such as the Effort Expenditure for Reward Task (EEfRT; Treadway et al., 2009) or hand-grip exertions, both of which have been used in ADHD populations (e.g., Addicott et al., 2019; Winter et al., 2019). In contrast, mental effort tasks typically modify existing cognitive paradigms to offer a choice between two effort-level options, such as the Cognitive Effort Discounting Task (Westbrook et al., 2013) or the Demand Selection Task (Kool et al., 2010). These, too, have been used in ADHD populations (e.g., Mies et al., 2019). The behavior patterns seen in effort preference paradigms allow for inferences about the conscious experience of effort as participants are expected to prefer less aversive tasks.

In the present study, we conduct a scoping review (Munn et al., 2018) of studies that assess the experience of mental effort in individuals with ADHD, either through direct self-report or effort preference paradigms. Furthermore, the present review includes studies on the experience of both physical and mental effort in individuals with ADHD, given that it is unclear whether the experiences differ or overlap.

Studies that employed either an effort preference paradigm (physical or mental) or direct self-reports of the conscious experience of effort while engaged in a demanding task (physical or mental) were included. Furthermore, studies must have used a measure of ADHD symptomatology or included a diagnosed ADHD sample. Studies that did not assess the experience of effort through self-report or effort preferences were excluded. We included all age ranges in our search to characterize the literature thoroughly. We systematically reviewed the literature to identify, appraise, synthesize, and identify gaps in the literature on the experience of effort in individuals with ADHD across the lifespan.

## 2 Method

Following the Joanna Briggs Methodology for Scoping Reviews (Joanna Briggs Institute, 2015), a scoping review was conducted to identify published studies concerning the experience of effort in ADHD. This review was formatted per the Preferred Reporting Items for Systematic Reviews and Meta-Analyses extension for Scoping Reviews (PRISMA-ScR; Tricco et al., 2018).

**Figure 2:**
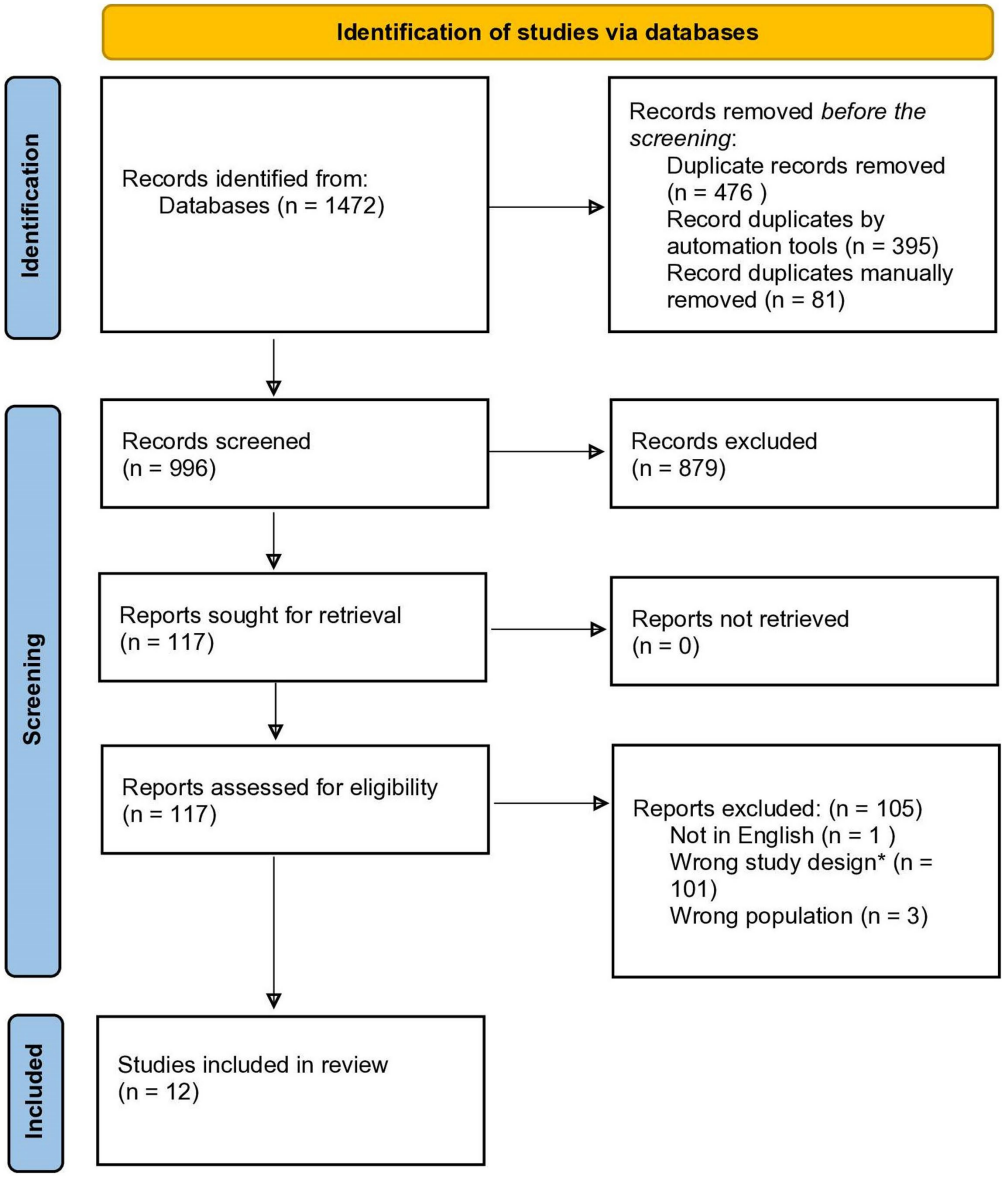
A model of the facets of the experience of mental effort. There are distinct cognitive and affective processes underlying these experiential aspects of using mental effort. There are also additional and important aspects of mental effort not included in this figure such as metacognitive processes.

A preliminary search was conducted to evaluate the volume of literature available and to determine if a review on the topic had been conducted. Few articles on the conscious experience of effort in ADHD were identified, and therefore, no publication date restrictions were set. A second search of PsycINFO (OVID), PsycINFO (ProQuest) and PubMed was conducted to gather the relevant literature. The search terms included (“ADHD” or “Attention-Deficit/Hyperactivity Disorder”) and (“effort” or “mental effort” or “cognitive effort” or “physical effort” or “mental load” or “cognitive load” or “workload” or “cognitive workload” or “task load” or “mental work” or “work”). The first two terms were chosen to capture studies referencing ADHD. The remaining terms were chosen because they aligned with the most common terminology in previous studies on the topic identified in the preliminary search. No limits were applied to the search within the three selected databases. All studies included in the review must have met the following inclusion criteria: All studies must be empirical and published in a peer-reviewed journal. Studies were required to be written and published in English before February 14, 2023, when the final search was conducted. The studies were required to include a group with ADHD or a measure of ADHD symptomatology. The conscious experience of effort must have been measured either by self-report in conjunction with a behavioral task (e.g., educational, cognitive, or physical) or a lab-based effort preference paradigm (cognitive or physical). Studies that did not measure the conscious experience of effort in one of those capacities were excluded. Therefore, studies that only included physiological indices of effort (e.g., pupillary, skin conductance, heart rate) in the absence of self-report or effort preferences were excluded. Only articles published in English were included in the review. The PRISMA-ScR flowchart is presented in Figure 2.

### 2.1 Search results

Potentially relevant publications, identified from the search of the three databases, were imported in abstract form into Covidence (www.covidence.org), a screening and data extraction tool used to manage the results at each screening stage. Given the broad search criteria and terms, the search initially generated 1,472 references, from which 476 duplicates were removed. After removing duplicate articles, two trained reviewers independently screened the 996 titles and abstracts identified through the selected databases, following the JBI guidelines. Both reviewers (DW and SGM) were students in psychology with background knowledge of the topic and were thoroughly trained on the pre-defined inclusion criteria for the review. When five disagreements were encountered, DW and SGM met to discuss the article(s) the reviewers disagreed about. Once a thorough discussion was had, a decision to include or exclude the article was finalized. Thirty-five of the 996 articles were discussed throughout the article review process, and a consensus was reached. The search yielded many results that did not match the inclusion criteria but were picked up because of the term “effort” in the abstract. For most of these articles, effort was used to describe the ‘efforts’ of researchers. For example, these articles described “continuous efforts” to address or establish areas of research. Thus, the conscious experience of effort was not the focus of the research. Accordingly, DW and SGM excluded 879 at the abstract screening stage and identified 117 articles for full-text screening.

Following the abstract screening, DW proceeded with the full-text review of the identified articles (*n* = 117). Since only one reviewer (DW) reviewed the full texts of the selected articles, the inclusion of some articles may have been biased. To help control this bias, SGM screened a random sample of 20% of the *n* = 117 articles reviewed at this stage. Based on the guidelines from Altman (1999) and adapted from Landis and Koch (1977), the random sample screened by SGM achieved substantial agreement, *κ* = 0.623. The reviewers discussed the two disagreements, and a consensus was reached where one of the two papers would be included in the study. After discussion, Cohen’s Kappa reached a near-perfect agreement, *κ* = 0.830. Twelve articles met the inclusion criteria for the review, while 105 studies were excluded. Among the excluded articles at the full-text stage, 101 were omitted due to inappropriate study design (i.e., did not measure the conscious experience of effort through self-report or preference paradigm), three did not include participants with ADHD, and one was excluded as it was not published in English.

### 2.2 Data charting process

Following the full-text review, DW charted data from the included articles (*N* = 12) to gather study information. The information gathered included title, author(s), year of publication, the definition of effort employed in the study, ADHD measures or the confirmation of diagnosis, ADHD medication, sample size, mean age, age range, gender, language status, measures of effort, tasks and self-report measures used. Additionally, data charting for each article included the following information: a summary of general findings and findings related to the conscious experience of effort. Each article was examined for the above information. Once this was completed, each article was reviewed a second time by the same reviewer and a third time by SGM to ensure all relevant information was reported correctly. Following the charting process, DW and SGM sought to identify patterns in the results. First, each study was categorized based on whether the study investigated mental or physical effort. Then, studies were grouped based on the definition of effort employed in the study. Finally, studies were clustered according to the ADHD measures employed. Patterns in the charting produced results from which conclusions could be drawn. The qualitative findings from this review are presented here.

## 3 Results and discussion

The scoping review identified *N* = 12 relevant studies, which were selected based on the predefined inclusion and exclusion criteria. A summary table is available in Supplementary material Table S1.

### 3.1 Variations in the definitions of effort

The definition of effort demonstrated considerable divergence among the 12 studies examined. These variations extended beyond distinctions between physical or mental effort. Moreover, the definition of effort employed by authors were often ambiguous and non-explicit, requiring DW and SGM to infer definitions from how researchers used the term.

Conceptualizing effort as related to, or a component of, motivation was the most common understanding of effort (Hoza et al., 2001; Fritz and O’Connor, 2016; Mies et al., 2018, 2019; Addicott et al., 2019; Winter et al., 2019; Brown et al., 2020). Relatedly, some studies did not emphasize motivation but focused on agentic ideas like “how hard I tried” (Mahon et al., 2012; Lewandowski et al., 2015). One study conceptualized mental effort as the total amount of controlled cognitive processing (Brown et al., 2020). Thus, notwithstanding the differences between motivation and volition, which is explored in the general discussion section, 75% of the studies reviewed employ a definition of effort that is in line with the notion of volitionally exerted effort (Hoza et al., 2001; Mahon et al., 2012; Lewandowski et al., 2015; Fritz and O’Connor, 2016; Mies et al., 2018, 2019; Addicott et al., 2019; Winter et al., 2019; Brown et al., 2020).

The second most common understanding of effort was to conceptualize effort as something that is activated or elicited by the demands of the task. For example, Johnstone et al. (2010) posited that effort encompasses arousal and activation, required to meet task demands, which are heavily determined by cognitive load and task difficulty, referring to a “how much the task activated me” understanding of mental effort. Neef et al. (2005) viewed mental effort like Johnstone et al. (2010), defining it as the response elicited by a task which is required for efficient task completion. Thus, taken together, 16.7% of studies defined effort in line with the conceptualization of effort as task elicited.

Lastly, one study did not define effort as task-elicited or volitionally exerted; instead, it focused on the affective component of effort. Namely, Hsu et al. (2017) conceived effort as the subjective experience of feeling burdened or taxed during task completion. This definition borrows some elements of a task-elicited definition of effort but emphasizes the affective component of effort.

To summarize, there was considerable variation in the conceptualizations of effort across the 12 studies. Most of the studies employed a definition aligned with a volitionally-exerted conceptualization of mental effort, but a few defined mental effort as task-elicited. As a result, it is difficult to articulate coherent generalizations across the studies that employ different definitions of effort. Greater clarity and consistency in the conceptualization of effort is needed to understand the conscious experience of effort in ADHD.

### 3.2 Self-report effort findings

Twenty-five percent of studies (*n* = 3 of 12) used a multi-faceted, self-report measure of the conscious experience of effort, which included an affective component (Hoza et al., 2001; Hsu et al., 2017; Mies et al., 2019).

Hoza et al. (2001) explored evaluations of perceived success, frustration, projected future performance, and task enjoyment of boys with and without ADHD while completing word puzzles with pre-determined success or failure outcomes (Hoza et al., 2001). This study found that the ADHD group endorsed feelings of frustration more than the control group in a failure task condition. Moreover, Hoza et al. (2001) found that compared to the control group, ADHD boys in the failure condition endorsed effort (defined as volitionally exerted) less as a reason for failure and instead ascribed failure to task difficulty and luck. Overall, compared to the control group, ADHD boys expressed more irritation and less hope about their future performance (Hoza et al., 2001).

Mies et al. (2019) did not find group differences in subjective ratings of effort, measured with the NTLX (defined as the energy needed to meet task demands, related to motivation—akin to volitionally-exerted effort), after completing an n-back working memory task, which requires participants to decide if a stimulus in a sequence matches a stimulus presented *n* items ago. In this study, boys aged 12–17 years were asked to complete different task difficulty levels, presented in increasing task difficulty (1-, 2-, 3-, 4-, 5-, and 6-back), and then rate their retrospective conscious experience using the NTLX after each task difficulty level. Although the ADHD group reported more frustration after completing the cognitive task than the control group, this difference was not significant. Notably, with increasing difficulty levels, the ADHD group reported less retrospective frustration levels as the task went on (Mies et al., 2019).

Hsu et al. (2017) found that an adult ADHD-probable group reported higher levels of in-the-moment mental effort (defined as the feeling of being taxed, akin to task-elicited effort) and discomfort during the Sustained Attention to Response Task (SART), which requires participants to withhold behavioral responses to infrequent target stimuli. Moreover, the researchers found that the ADHD-probable group reported higher levels of mental effort and discomfort at the peak and the end of the task (Hsu et al., 2017). When differences in cognitive performance were controlled for, these differences in levels of mental effort and discomfort in the moment, at the peak, and the end of the task remained (Hsu et al., 2017).

These results suggest that multi-faceted approaches to the self-reported experience of effort may offer a more comprehensive assessment of the experience and better determine when and where differences exist between groups with ADHD and controls. In all studies using such measures, the ADHD group showed more affective distress than the control group, although this difference was not significant in one study (Mies et al., 2019). These results further validate the notion that diagnostic criterion (f) is an affective one and should be studied accordingly.

Of the studies included in the review, 41.6% employed (*n* = 5 of 12) a single item to measure effort through self-report. Brown et al. (2020) explored how different instructions, specifically multimedia instruction with and without subtitles, influence the mental effort experience in adults. The researchers found that greater degrees of self-reported mental effort (defined as the amount of controlled cognitive processing akin to volitionally exerted effort) were associated with higher levels of ADHD symptomatology (Brown et al., 2020). Fritz and O’Connor (2016) also explored the relationship between ADHD symptomatology and self-reported willingness to exert volitional effort in an adult population. The researchers found that following moderate exercise, there was a significant increase in willingness to complete mental tasks in an adult sample high in ADHD symptomatology. Johnstone et al. (2010) explored how stimulus degradation impacted self-reports of effort (i.e., task-elicited effort) in ADHD. They found that the ADHD group reported more effort in a 0% stimulus degradation condition but less effort in a 60% stimulus degradation condition than controls. This result from Johnstone et al. (2010) suggests that task demands may have complex non-linear influences on the experience of mental effort, depending on ADHD status. One study did not find differences in the rate of perceived exertion (i.e., volitionally exerted effort) between a group with ADHD and controls while completing physical exercise (Mahon et al., 2012). Lastly, one study did not report their effort findings despite measuring perceived effort (i.e., volitionally exerted effort) during a reading comprehension test (Lewandowski et al., 2015). Taken together, these findings suggest that single-item scales may be able to pick up on meaningful differences in the conscious experience of effort between ADHD groups and controls; however, studies using single-item effort scales do not capture all the components of the conscious experience of effort and have not examined affective qualities.

In summary, the studies employing self-report measures of the conscious experience of effort produced varying results. These mixed results may stem from different definitions of effort and different measurement tools. Existing self-report measures of mental effort are limited in some respects. First, four of the studies that used self-report employed retrospective measures, which require participants to reflect on their experience of mental effort after finishing a task. Although retrospective ratings are important and relevant for regulating future behavior, they are inaccurate proxies of the experience during a task, which may predict persistence with cognitively demanding activities. Biases, such as the peak-end effect, describe a phenomenon in which participants’ retrospective judgments of an experience are heavily influenced by their ratings of the experience at its peak and end (Redelmeier and Kahneman, 1996). Previous studies, including Hsu et al. (2017), have found peak-end effects in mental effort ratings (e.g., Hsu et al., 2017; Bambrah et al., 2019). The use of retrospective and in-the-moment self-report measures of the experience of mental effort may explain some of the disparate results. Future studies should use both retrospective and in-the-moment measures of the experience of mental effort to better understand how the experience during and after a task influences willingness to engage in and persist with cognitively demanding tasks. Secondly, the use of single-item scales fails to capture the important facets of the experience of mental effort, while also offering limited psychometric properties. Future studies should use multi-faceted scales to better capture the experience of mental effort.

### 3.3 Effort preference paradigm findings

Nearly 42 % (41.5%) of total studies (*n* = 5 of 12) employed a preference paradigm (Neef et al., 2005; Mies et al., 2018, 2019; Addicott et al., 2019; Winter et al., 2019). One study used an effort preference paradigm in addition to a multi-faceted self-report measure (Mies et al., 2019). Of the studies employing a preference paradigm, 60% (3 out of 5) used a physical effort preference paradigm, and 40% (2 out of 5) used a mental effort preference paradigm. In these preference paradigms, preferences are operationalized as the proportion of high-task demand choices made compared to low-task demand choices. Where more low-task demand choices are made, greater ‘effort aversion’ is observed. Therefore, unlike self-report measures, the experience of effort is operationalized indirectly in terms of behavior. One study (Mies et al., 2019) used a multi-faceted retrospective self-report measure in conjunction with a cognitive task, followed by an effort preference paradigm; however, these operationalizations of effort were employed independently.

#### 3.3.1 Physical effort preference findings

Of the studies, 25% (*n* = 3 of 12) used a physical effort preference paradigm. Addicott et al. (2019) explored how ADHD medications impact willingness to engage in physically effortful tasks, where effort was defined as a component of motivation (i.e., akin to volitionally exerted effort). This study found an interaction between group and drug administration, such that after the administration of stimulant medications, there was a greater increase in the average number of high-effort selections for reward between the drug and placebo conditions in the adult ADHD group than in the control (Addicott et al., 2019). However, the researchers did not find any group differences or differences between drug conditions in the number of trials completed or the ratio of high-effort trials selected (Addicott et al., 2019).

Similarly, Winter et al. (2019) did not find a significant group difference between children with and without ADHD in the number of high-effort choices made during a physical effort hand dynamometer task. In this task, participants were asked to choose between four trial types: high effort-high reward, high effort-low reward, low effort-high reward, and low effort-low reward (Winter et al., 2019). The results showed no group differences between the number of high effort-high reward choices made; however, the ADHD participants failed to successfully recruit the physical force needed to meet the threshold for the high-effort selection more often than controls (Winter et al., 2019). Both groups showed a similar tendency to choose higher effort for higher rewards, but the ADHD group was less successful in executing high-effort choices (Winter et al., 2019).

Mies et al. (2018) used a hand dynamometer task in which participants chose between a small reward for little physical effort (15% of their maximum hand grip effort) or a larger fixed reward for more effort (90% of their maximum effort). In addition to preferences, Mies et al. (2018) included measures of valence and arousal while implementing the effort preference paradigm. The researchers did not find differences in physical effort preferences in adolescents with ADHD compared to the control group or observe group differences in self-reported valence and arousal ratings (Mies et al., 2018).

The studies that used physical preference paradigms in conjunction with rewards did not reveal differences in the experience of effort between ADHD and control groups. Although the research is limited, this finding suggests that there may not be physical effort aversions in ADHD. It is important to note that the DSM criteria for ADHD describe the dislike or aversion to sustained mental effort rather than physical effort (APA, 2022).

#### 3.3.2 Mental effort preference findings

Two studies (16.7%) examined the experience of effort in ADHD using mental effort preference paradigms. For example, Mies et al. (2019) employed the Cognitive Effort Discounting Task (COGED), a mental effort preference paradigm, with male adolescents who were asked to repeatedly choose between doing an easier n-back task (1-back) for a smaller, fixed monetary reward and a more arduous n-back task (2-, 3-, 4-, 5-, and 6-back) for a larger, variable monetary reward. The researchers did not find group differences in effort preferences on the COGED task (Mies et al., 2019). Neef et al. (2005) explored children’s choices to complete more vs. less challenging math problems. After completing the task, the children in the study were asked to rate the factors that influenced their choices. The researchers did not report the choice results but found that the ADHD group reported their choices were most influenced by the immediacy and quality of the reinforcers offered rather than by the difficulty of the question (Neef et al., 2005). However, the ability to draw conclusions from this study is limited, given the researchers did not report the findings on behavioral choice. In conclusion, only two studies used a mental effort preference paradigm, and only one reported the preference findings. All the effort preference paradigms included rewards. Further research is needed to see if differences are observed between ADHD and control groups when using mental effort preferences paradigms.

Operationalizing the experience of mental effort in terms of behavioral choices in effort preference paradigms presents some challenges. Namely, existing effort preference paradigms used to study individuals with ADHD have all included rewards. The inclusion of rewards makes it difficult to isolate effects that are specific to the experience of effort. Indeed, in ADHD, suboptimal reward processes have been proposed as a primary cause of dysfunction in ADHD (e.g., Sonuga-Barke et al., 1994; Sagvolden, 1996; Sagvolden et al., 1998). Thus, the presence of rewards may confound the study of the conscious experience of effort in ADHD and leave open the question of effort aversion in ADHD. Ideally, research studies should seek to disentangle effects resulting from suboptimal reward processes versus those resulting from an aversion to effort. For example, future studies could use a mental effort preference paradigm that controls the presence or absence of rewards to determine if independent effort aversion and reward effects are observed in ADHD.

Additionally, there is a need to explore how different kinds of cognitive tasks may influence the experience of mental effort, as highlighted by Mies et al. (2019). For example, Mies et al. (2019) demonstrated that increasing working memory load [i.e., computational complexity (Mulder, 1986)] did not lead to group differences. However, tasks that require sustained attention or vigilance [i.e., compensatory control (Mulder, 1986; Fairclough and Mulder, 2012)] may give rise to different experiences, and these differences might interact with ADHD status. Indeed, Hsu et al. (2017) used a task that required compensatory control (the SART) and found that individuals high in ADHD symptoms experienced more significant discomfort and reported more effort (i.e., the experience of being taxed) than the control group and that these differences in the experience of effort remained even when matched on task performance and cognitive ability. Given that these tasks require compensatory control and require individuals to exert effort volitionally, they may induce a different experience of effort compared to computationally complex tasks, where effort is more effectively elicited by task demands. Compensatory control tasks, like the continuous performance task (Rosvold et al., 1956), have a long history of being used to explore cognitive deficits in ADHD (e.g., Riccio and Reynolds, 2003; Huang-Pollock et al., 2012); however, patterns of poor performance have primarily been explained by executive functioning (e.g., Barkley, 1997) or physiological effort regulation deficits (e.g., Sergeant, 2000). In contrast, poor performance may partly stem from the aversive experience of volitionally exerting effort under conditions where the task does not elicit sufficient activation.

### 3.4 Study demographics

#### 3.4.1 Gender

Twenty-five percent (3 of 12) of the studies focused exclusively on mental effort in male individuals (men/boys) with ADHD. Hoza et al. (2001) found that ADHD boys expressed more frustration and less hope about their future performance on word puzzles and endorsed less effort (i.e., volitionally exerted effort) and ascribed failure to task difficult and luck compared to controls. Mies et al. (2019) did not find differences in the effort experience between adolescent males with and without ADHD. Finally, Fritz and O’Connor (2016) found that the willingness to do mentally effortful tasks increased, and mood improved, after 20 min of moderate exercise in an adult male with heightened ADHD symptoms sample. Thus, research into the experience of mental effort, in male populations is mixed and may depend on the age of the participants and the type of activity. The remaining nine studies included two genders (women and men). The absence of literature focused explicitly on the experience of effort in female and gender nonbinary individuals with ADHD is a critical gap in the literature.

Much of the existing research on ADHD has focused on boys and men (Hartung and Widiger, 1998). Indeed, gender differences in ADHD are poorly understood (Hasson and Fine, 2012), which can have downstream effects on diagnosis and treatment. Boys are more likely to be diagnosed and treated for ADHD than girls, which may lead to consistent under-identification and underdiagnosis in the female population (Quinn and Madhoo, 2014; Visser et al., 2014). Studies investigating gender differences in ADHD have found that girls diagnosed with ADHD show more inattentive symptoms when compared to boys with ADHD (Biederman et al., 2002; Biederman and Faraone, 2004). Since the mental effort diagnostic criterion is an inattentive symptom (APA, 2022), exploring the gender differences in the conscious experience of effort may be important for understanding ADHD in women and girls. Moreover, none of the studies in the present review included gender-fluid or gender-nonconforming individuals.

#### 3.4.2 Age, racial and ethnic identity, and socioeconomic status

Thirty-three percent of the reviewed literature (*n* = 4 of 12) specifically examined adults with ADHD, including participants aged 18–65 years, with most of these studies focused on young adult populations (ages 18–25) (Fritz and O’Connor, 2016; Hsu et al., 2017; Addicott et al., 2019; Brown et al., 2020). However, given the different approaches employed in these studies, it is difficult to synthesize the findings. It may be the case that the type of task (i.e., mental versus physical; see descriptions above of Hsu et al., 2017; Addicott et al., 2019; Brown et al., 2020) influences the type of results observed in adult populations. Overall, the results suggest that within adult samples, those with ADHD may experience mental effort differently than controls, such that it is more aversive for those with ADHD, but further research is needed to clarify these patterns of results.

Twenty-five percent (*n* = 3 of 12) of the studies focused on adolescents, comprising individuals between 12 and 17. The studies exploring the experience of effort in adolescents did not find group differences between participants with ADHD and controls (Lewandowski et al., 2015; Mies et al., 2018, 2019).

The remaining five studies (41.7%) concentrated on children, involving participants aged up to 14. Of these five studies, one did not find group differences in the conscious experience of effort (Mahon et al., 2012), two focused on attributions (i.e., causes of performance) and reinforcer preferences (i.e., type, rate, and quality of reinforcements) (Hoza et al., 2001; Neef et al., 2005), and two studies reported group differences depending on what was asked of the participants (Johnstone et al., 2010; Winter et al., 2019). Together, these results may suggest that children with ADHD may experience effort differently than those without ADHD. Specifically, the results suggest that children with ADHD may struggle with effort regulation due to under-engaging tasks and that this may depend on task characteristics.

Overall, the findings from this review suggest that age may influence the pattern of results related to the conscious experience in effort in ADHD. The literature was mixed in both adult and child samples, whereas there were no observed differences between individuals with ADHD and controls in adolescent samples. Lastly, there were no studies of individuals with ADHD in older adult populations over 65 years.

The studies reviewed contained limited information regarding race, ethnicity, and socioeconomic status. Only two studies reported the racial demographics of the participants included in the study (Neef et al., 2005; Lewandowski et al., 2015), and only one study reported the participants’ socioeconomic background (Lewandowski et al., 2015). Previous research has shown disparities in recognizing ADHD symptoms among racialized groups, predominantly African-American and Latino individuals (Coker et al., 2016; DuPaul, 2020). This disparity is likely related to the underdiagnosis and undertreatment of ADHD among racialized individuals. Therefore, the present review echoes previous calls for the reporting and justification of participant demographics, as this may lead to better recognition and identification of ADHD symptoms in diverse individuals and may help ameliorate existing racial disparities (Roberts et al., 2020; Dupree and Kraus, 2022).

### 3.5 ADHD diagnosis

Seven studies (58.3%) employed a formal diagnosis of ADHD based on DSM criteria. This approach involved obtaining relevant documentation from the participants or having qualified and licensed clinicians conduct comprehensive assessments, including structured or semi-structured interviews, to establish the ADHD diagnosis before their inclusion in the study (Hoza et al., 2001; Johnstone et al., 2010; Lewandowski et al., 2015; Mies et al., 2018, 2019; Addicott et al., 2019; Winter et al., 2019). Four of the studies using a formal ADHD diagnostic procedure (Mies et al., 2018, 2019; Addicott et al., 2019; Winter et al., 2019) did not find group differences between participants with ADHD and controls, and one study did not report effort findings (Lewandowski et al., 2015). However, two studies found that self-reported experiences of effort (Johnstone et al., 2010) or effort attributions (Hoza et al., 2001) depended on task conditions, described above.

Three studies (23%) relied on self-report measures to measure ADHD symptomatology and assign participants to groups based on cut-off scores (Fritz and O’Connor, 2016; Hsu et al., 2017; Brown et al., 2020). These self-report measures included Barkley’s Self-Report Scale (Barkley, 2011) and the Adult ADHD Self-Report Scale (ASRS; Kessler et al., 2005; Ustun et al., 2017). One study found a significant association between ADHD symptoms and self-reported effort, such that ADHD symptoms accounted for 7% of the variance in self-reported mental effort (Brown et al., 2020). Fritz and O’Connor (2016) employed a within-subjects design. They found that acute moderate-intensity exercise increased motivation to complete cognitive tasks in men who met the ASRS cut-off score. Finally, Hsu et al. (2017) found that in-the-moment effort ratings significantly predicted retrospective discomfort ratings following the completion of a cognitive task in an ADHD-probable group (based on ASRS cut-off scores) but not in the control group.

Although most studies reported how they established an ADHD diagnosis, or measured ADHD symptomatology, two studies did not describe how the ADHD diagnosis was confirmed but stated that the participants “had ADHD” or met diagnostic criteria (Neef et al., 2005; Mahon et al., 2012).

In summary, studies that used a formal ADHD diagnosis obtained mixed finings at the group level; whereas, studies employing self-report measures generated more consistent associations between the experience of effort and ADHD symptomatology. However, results from studies using self-report measures of ADHD should be cautiously interpreted. A recent systematic review showed that self-report measures of ADHD symptomology have exceedingly high false positive rates, reducing their clinical utility (Harrison and Edwards, 2023). Using self-report measures and semi-structured interviews without additional assessment measures may lead to the overidentification of individuals with ADHD (Harrison and Edwards, 2023). Altogether, these findings suggest considerable variability in how researchers diagnosed and/or measured ADHD. Therefore, future research should include more rigorous assessments of ADHD symptoms and diagnosis to clarify this population’s experience of effort, especially given avoidance or dislike of sustained mental effort is included as a diagnostic criterion.

## 4 General discussion

Considering the inclusion of sustained mental effort ‘avoidance’ and ‘dislike’ among the diagnostic criteria of ADHD, and given the critical role that the experience of mental effort plays in regulating cognition (e.g., Hockey, 2011; Kurzban et al., 2013), it was important to conduct a comprehensive review of studies exploring the experience of effort in ADHD. The present review identified several gaps in the research. To begin with, we were only able to identify 12 studies that assessed the experience of effort in ADHD, and thus, significantly more research is needed in this area. Below we articulate several recommendations to guide future research.

The results show significant variability in, and under-specification of, the definition of mental effort. Based on a review of the theoretical and empirical work, we propose three distinct aspects of mental effort: task-elicited and volitionally exerted cognitive factors, and affective factors, each of which can be consciously experienced. Future studies investigating the experience of mental effort should be more precise as to how mental effort is being conceptualized. We recommend that future research include all facets of mental effort.

None of the research in the present review measured the perception of task-elicited mental effort, volitionally-exerted mental effort, and the feeling of engaging in mental effort together in one study. Many studies measured volitionally exerted effort, a few assessed task-elicited effort, and even fewer assessed the associated affective experience. According to Sergeant’s theory, individuals with ADHD may not be sufficiently activated or aroused by cognitive tasks and, therefore, may have to exert more effort volitionally to complete a task successfully (Sergeant, 2000). Understanding the factors that sufficiently arouse or activate individuals with ADHD may also give insight into the experience of hyperfocus in ADHD. Hyperfocus refers to a heightened state of focused attention and has been reported as a common experience for individuals with ADHD relative to controls (Hupfeld et al., 2019; for a review of hyper-focus, see Ashinoff and Abu-Akel, 2021).

Barkley’s executive functioning theory of ADHD proposes that one of the core deficits in ADHD is self-regulation of affect, motivation, and arousal (Barkley, 1997). This theory posits that impairment in several domains of life stems from the struggle of individuals with ADHD to initiate and maintain motivation, arousal, and affect regulation (Barkley, 1997). Evidence for motivational deficits in ADHD stems from studies that have shown that individuals with ADHD have reduced dopamine receptors in the nucleus accumbens, an area of the brain thought to be implicated in motivation (Volkow et al., 2011), or by measuring the reactions of individuals to behavioral contingencies, rewards, and consequences (e.g., Carlson and Tamm, 2000; Slusarek et al., 2001). However, currently, there is limited evidence of motivational deficits in ADHD (for review, see Smith and Langberg, 2018). It is also important to distinguish between motivation and volition. Motivation refers to the desire to engage in an activity, while volition refers to the capacity to effectively move toward a goal (Eccles and Wigfield, 2002). As Winter et al. (2019) showed, in ADHD, motivation is related to, but not the same as, the volition needed to complete a task successfully. The use of self-report and effort preference paradigms can measure motivation (i.e., willingness to engage in) and volition (i.e., capacity to move toward a goal) through successive choices. More specifically, using effort preference paradigms, the choices of successive trials and successful (or unsuccessful) completion of those trials will measure motivation (choice) as well as volition (capacity to complete). Additionally, administration of in-the-moment self-report measures can better clarify the roles of motivation and volition on performance. Thus, directly measuring volitionally exerted effort in ADHD may help clarify the role of volition in effortful tasks, while effort preference paradigms can clarify the role of motivation.

Theories of effort (e.g., Kurzban et al., 2013) highlight how affect regulates cognition. Therefore, measuring the feeling of engaging in mental effort is critical to understanding persistence with activities. It may be the case that emotional processes contribute to performance on cognitive tasks. Emotional regulation difficulties are observed in ADHD (Martel, 2009; Soler-Gutiérrez et al., 2023). Critically, these difficulties have typically been explained and studied in interpersonal contexts (for review, see Soler-Gutiérrez et al., 2023), and very little research has explored how emotion regulation influences the conscious experience of mental effort or how emotions influence performance on cognitive tasks (Groves et al., 2020). For example, it may be that even with the same level of ability and the same level of performance, individuals with ADHD might experience mental effort as being more aversive. This affective experience may, in turn, predict choices and persistence with cognitively demanding tasks.

A similar line of inquiry has been explored by researchers investigating delay aversion, finding that individuals with ADHD do not necessarily have poor impulse control in delay-rich environments, but rather, they experience delays to be more aversive (Sonuga-Barke et al., 1992; Marco et al., 2009; Sonuga-Barke et al., 2010). Similarly, research is needed to explore how the feeling of engaging in mental effort influences performance on cognitive tasks in ADHD and if these feelings are related to, or distinct from, more general emotion regulation difficulties observed in ADHD. The present study provides evidence for a pattern of emotional reactions (i.e., frustration) to mentally effortful tasks in ADHD, pointing to a potentially exciting area of research. However, without direct measurement of all facets of the experience of effort, it is unclear where struggles with effort regulation in ADHD lay. Therefore, future studies should include self-report measures that assess all three components to better describe the conscious experience of effort in ADHD.

### 4.1 Limitations

There are several limitations to the present review. First, given that one reviewer was responsible for determining article inclusion, the selection of some articles may be biased. Second, only twelve studies were included in the final review, with many studies using different methods, populations, and conceptualizations of mental effort. Moreover, many studies relied on self-reported ADHD symptoms as opposed to rigorous diagnostic procedures. As a result, conclusions drawn from this review about the experience of mental effort in ADHD are limited. Third, while some authors clearly defined and operationalized effort in their studies, others did not, and included very little theory or conceptualization of effort. As a result, we had to infer the conceptualization of effort being offered by the authors. More specifically, we classified existing conceptualizations of mental effort into the taxonomy we established from our consideration of existing theories of mental effort (see Figure 1). However, there may be other ways of coherently and usefully organizing existing empirical findings related to the experience of effort in ADHD. Lastly, the present review is focused only on the experience of mental effort in ADHD, without consideration of comorbidities. As previous research has shown, there is a high rate of comorbidity with ADHD, most commonly anxiety and behavioral disorders (e.g., Katzman et al., 2017; Mohammadi et al., 2021). These comorbidities may also shape the experience of mental effort (Wolpe et al., n.d.). Indeed, recent researchers have conceptualized mental effort as an important transdiagnostic phenomenon that cuts across several diagnostic categories (Patzelt et al., 2019). Nevertheless, given that struggles with avoidance or dislike of sustained mental effort is a diagnostic criterion of ADHD, it is essential to describe how individuals with ADHD, with or without comorbid disorders, experience mental effort. Future reviews could explore the experience of mental effort more broadly, across different forms of psychopathology, to understand the concept better.

### 4.2 Conclusion

Findings of the current review suggest individuals with ADHD might not be sufficiently activated by tasks and, therefore, may require additional external factors to up-regulate arousal and activation and/or may need to volitionally exert more effort to compensate for the insufficient levels of task elicited effort. For example, Mies et al. (2018) found that increasing working memory demands resulted in less frustration among individuals with ADHD, possibly because the task elicited more activation which reduced the ADHD participants’ need to volitionally exert more effort. Similarly, Fritz and O’Connor (2016) found that rigorous physical activity resulted in increased willingness to volitionally exert effort, and Addicott et al. (2019) found that stimulant medication had a greater impact on ADHD participants willingness to volitionally exert effort compared to non-ADHD participants. In turn, individuals with ADHD may feel worse while engaging in mental effort, as evident in Hsu et al. (2017) finding of increased feelings of distress in the ADHD probable group despite comparable performance and ability. Finally, the increased distress associated with volitionally exerting effort may, in turn, result in disengagement from the task and less willingness to do the task again in the future (Hsu et al., 2017). However, it is difficult to determine where precisely the differences between individuals with and without ADHD lie without exhaustively measuring the different aspects of mental effort while participants engage in different types of cognitive tasks.

## Data availability statement

The original contributions presented in the study are included in the article/supplementary material, further inquiries can be directed to the corresponding author.

## Author contributions

DW: Writing – review & editing, Writing – original draft, Methodology, Conceptualization. SM: Writing – review & editing, Methodology. JE: Writing – review & editing, Conceptualization.
