# Supplementary material for: The experience of effort in ADHD: a scoping review
Source: Front Psychol. 2024 Jun 3;15:1349440. doi: 10.3389/fpsyg.2024.1349440 (PMC11184226; doi:10.3389/fpsyg.2024.1349440)
Supplement: Supplementary file 1 [file Data_Sheet_1.PDF]

Table S1

Summary of Review Results

Publication	Mental or Physical Effort	Mental Effort Related Term	Definition	Facet of the Experience of Mental Effort	ADHD Measure	Participants	Effort Operationalization	Results
Brown et al., 2020	Mental	Mental effort	The total amount of controlled cognitive processing.	Volitionally exerted.	ADHD Symptoms	Young Adults Mixed Gender	Single-item self-report.	Higher levels of ADHD symptomatology were associated with greater degrees of self-reported mental effort.
Fritz & O'Connor, 2016	Mental	Motivation to perform mental work	A component of motivation, willingness to exert effort; important in decision making.	Volitionally exerted.	ADHD Symptoms	Young Adults Men	Single-item self-report.	Following moderate exercise, there was a significant increase in willingness to complete mental tasks for those high in ADHD symptomatology.
Hoza et al., 2001	Mental	Effort	The energy needed to meet task demands, influenced by motivational factors.	Volitionally exerted.	ADHD Diagnosis	Children Boys	Multi-faceted self-report.	The ADHD group endorsed feelings of frustration more than the control group in a failure task condition. The ADHD group in the failure condition endorsed effort

								less as a reason for failure.
Hsu et al., 2017	Mental	Mental effort	The experience of being burdened or taxed.	Affective aspect.	ADHD Symptoms	Young Adults Mixed Gender	Multi-faceted self-report.	The ADHD-probable group reported higher levels of in the moment mental effort and discomfort than the control group. The ADHD-probable group reported higher levels of mental effort and discomfort at the peak and end of the task than the control group. When performance on the task was controlled for, these differences remained.
Johnstone et al., 2010	Mental	Effort	The level of energy required to meet task demands, influenced by cognitive load/task	Task-elicited.	ADHD Diagnosis	Children Mixed Gender	Single item self-report.	The ADHD group reported more effort in a 0% stimulus degradation condition but less effort in a 60% stimulus

			difficulty and the regulation of arousal and activation.					degradation condition than controls.
Lewandowski et al., 2015	Mental	Effort	How hard I tried.	Volitionally exerted.	ADHD Diagnosis	Adolescents (ages 12-17) Mixed Gender	Single item self-report.	Did not report single-item self-report results.
Mies et al., 2019	Mental	Mental effort	The energy needed to meet task demands by regulating physiological state, or motivational factors.	Volitionally exerted.	ADHD Diagnosis	Adolescents (ages 12-17) Boys	Multi-faceted self report; mental effort preference paradigm.	No differences between ADHD group and controls in subjective ratings of effort. ADHD group reported more frustration after completing a cognitive task than the control group, but this difference was not significant.<br><br>No difference in effort preferences.
Neef et al., 2005	Mental	Response effort	The work required to complete task.	Task-elicited.	Unclear	Children Mixed Gender	Mental effort preference paradigm.	Results on behavioural choices not reported.

Addicott et al., 2019	Physical	Effort	A component of motivation, willingness to exert effort important in decision making.	Volitionally exerted.	ADHD Diagnosis	Young Adults Mixed Gender	Physical effort preference paradigm.	After administration of stimulant medication, there was a greater increase in the average number of high-effort selections for reward between the drug and placebo condition in the ADHD group than in the control group. No group differences in the number of trials completed or the ratio of high-effort to low-effort trials selected.
Mahon et al., 2012	Physical	Perceived exertion	How hard I tried.	Volitionally exerted.	Unclear	Children Mixed Gender	Single-item self-report.	No group differences in rating of perceived effort.
Mies et al., 2018	Physical	Effort discounting	The energy needed to meet task demands, related to motivational factors.	Volitionally exerted.	ADHD Diagnosis	Adolescents (ages 12-17) Mixed Gender	Physical effort preference paradigm.	No group differences in physical effort preferences.

Winter et al., 2019	Physical	Effort	Related to motivation and the recruitment of energy to successfully complete a task.	Volitionally exerted.	ADHD Diagnosis	Children Mixed Gender	Physical effort preference paradigm.	No group differences in the number of high effort-high reward choices made. ADHD group failed to successfully complete the high-effort selections more often than the control group.
